# Rationale and study design for an individualized perioperative open lung ventilatory strategy (iPROVE): study protocol for a randomized controlled trial

**DOI:** 10.1186/s13063-015-0694-1

**Published:** 2015-04-27

**Authors:** Carlos Ferrando, Marina Soro, Jaume Canet, Ma Carmen Unzueta, Fernando Suárez, Julián Librero, Salvador Peiró, Alicia Llombart, Carlos Delgado, Irene León, Lucas Rovira, Fernando Ramasco, Manuel Granell, César Aldecoa, Oscar Diaz, Jaume Balust, Ignacio Garutti, Manuel de la Matta, Alberto Pensado, Rafael Gonzalez, Mª Eugenia Durán, Lucia Gallego, Santiago García del Valle, Francisco J Redondo, Pedro Diaz, David Pestaña, Aurelio Rodríguez, Javier Aguirre, Jose M García, Javier García, Elena Espinosa, Pedro Charco, Jose Navarro, Clara Rodríguez, Gerardo Tusman, Francisco Javier Belda, on behalf of the iPROVE investigators (Appendices 1 and 2)

**Affiliations:** Anesthesiology and Critical Care Department, Hospital Clínico of Valencia, Av. Blasco Ibañez, 17, Valencia, CP: 46010 Spain; Anesthesiology and Critical Care Department, Hospital Germans Tries i Pujol, Carretera de Canyet s/n, 08916 Badalona, Spain; Anesthesiology and Critical Care Department, Hospital San Pau, Carrer de Sant Quintí, 89, CP: 08026 Barcelona, Spain; Intensive Care Department, Uppsala University Hospital, Suecia Akademiska Sjukhuset Uppsala University, CP: 75185 Uppsala, Sweden; FISABIO salud Pública, Av. Cataluña, 21, CP: 46020 Valencia, Spain; Anesthesiology and Critical Care Department, Hospital de Manises, Av. De la Generalitat Valenciana, Manises, CP: 46940 Spain; Anesthesiology and Critical Care Department, Hospital La Princesa of Madrid, Calle de Diego León, 62, CP: 28006 Madrid, Spain; Anesthesiology and Critical Care Department, Hospital General of Valencia, Av. De les Tres Creus, 2, Valencia, CP: 46014 Spain; Anesthesiology and Critical Care Department, Hospital Río Hortega of Valladolid, Calle Dulzaina, 2, Valladolid, CP 47012 Spain; Anesthesiology and Critical Care Department, Hospital La Fe of Valencia, Av. De Fernando Abril Martorell, 106, Valencia, CP: 46026 Spain; Anesthesiology and Critical Care Department, Hospital Clínic i Provincial of Barcelona, Carrer Villarroel 170, Barcelona, CP: 08036 Spain; Anesthesiology and Critical Care Department, Hospital General Gregorio Marañon of Madrid, Calle del Doctor Esquerdo, 46, Madrid, CP: 28007 Spain; Anesthesiology and Critical Care Department, Hospital Vírgen del Rocio of Sevilla, Av. Manuel Siurot s/n, Sevilla, CP: 41013 Spain; Anesthesiology and Critical Care Department, Complejo Hospitalario Juan Canalejo of La Coruña, Xubias, 84, La Coruña, CP: 15006 Spain; Anesthesiology and Critical Care Department, Hospital of León, C/ Altos de Nava s/n, Leon, CP: 24701 Spain; Anesthesiology and Critical Care Department, Hospital Vírgen de la Arraixaca of Murcia, Carretera de Madrid-Cartagena s/n, Madrird, CP: 30120 Spain; Anesthesiology and Critical Care Department, Hospital Miguel Servet of Zaragoza, Paseo Isabel la Católica, 1-3, Zaragoza, CP: 50009 Spain; Anesthesiology and Critical Care Department, Hospital Fundación of Alcorcón, Calle de Valdelaguna, 1, Alcorcón, CP: 28922 Spain; Anesthesiology and Critical Care Department, Hospital General of Ciudad Real, C/ Alisos, 19, Ciudad Real, CP: 13002 Spain; Anesthesiology and Critical Care Department, Hospital de Valme of Sevilla, Av. Bellavista s/n, Sevilla, CP: 41014 Spain; Anesthesiology and Critical Care Department, Hospital Ramón y Cajal of Madrid, Carretera de Colmenar Viejo Km 9, Madrid, CP: 28034 Spain; Anesthesiology and Critical Care Department, Hospital de Gran Canaria Dr. Negrín, c/ Barranco de la Ballena s/n, Negrin, CP: 35010 Spain; Anesthesiology and Critical Care Department, Hospital of Galdakano, Barrio Labeaga s/n, Galdakano, CP: 48960 Spain; Anesthesiology and Critical Care Department, Complejo Hospitalario Juan Ramón Jimenez of Huelva, Ronda exterior norte, s/n, Huelva, CP: 21005 Spain; Anesthesiology and Critical Care Department, Hospital Puerta de Hierro of Majadahonda, C/ Manuel de Falla, 1, Majadahonda, CP: 28222 Spain; Anesthesiology and Critical Care Department, Hospital Nuestra Señora de la Candelaria of Santa Cruz de Tenerife, Carretera del Rosario, 145, Santa Cruz de Tenerife, CP: 38010 Spain; Anesthesiology and Critical Care Department, Hospital Son Espases of Mallorca, Carretera de la Valldemosa, 79, Mallorca, CP: 07120 Spain; Anesthesiology and Critical Care Department, Hospital General of Alicante, Pintor Baeza, 12, Alicante, CP: 03010 Spain; Anesthesiology Department, Hospital Privado de Comunidad Mar de Plata, Mar de Plata, Argentina

**Keywords:** Postoperative pulmonary complications, Open lung approach, Recruitment maneuvers, Positive end-expiratory pressure, Continuous positive airway pressure, Lung protective ventilation

## Abstract

**Background:**

Postoperative pulmonary and non-pulmonary complications are common problems that increase morbidity and mortality in surgical patients, even though the incidence has decreased with the increased use of protective lung ventilation strategies. Previous trials have focused on standard strategies in the intraoperative or postoperative period, but without personalizing these strategies to suit the needs of each individual patient and without considering both these periods as a global perioperative lung-protective approach. The trial presented here aims at comparing postoperative complications when using an individualized ventilatory management strategy in the intraoperative and immediate postoperative periods with those when using a standard protective ventilation strategy in patients scheduled for major abdominal surgery.

**Methods:**

This is a comparative, prospective, multicenter, randomized, and controlled, four-arm trial that will include 1012 patients with an intermediate or high risk for postoperative pulmonary complications. The patients will be divided into four groups: (1) individualized perioperative group: intra- and postoperative individualized strategy; (2) intraoperative individualized strategy + postoperative continuous positive airway pressure (CPAP); (3) intraoperative standard ventilation + postoperative CPAP; (4) intra- and postoperative standard strategy (conventional strategy). The primary outcome is a composite analysis of postoperative complications.

**Discussion:**

The Individualized Perioperative Open-lung Ventilatory Strategy (iPROVE) is the first multicenter, randomized, and controlled trial to investigate whether an individualized perioperative approach prevents postoperative pulmonary complications.

**Trial registration:**

Registered on 5 June 2014 with identification no. NCT02158923.

**Electronic supplementary material:**

The online version of this article (doi:10.1186/s13063-015-0694-1) contains supplementary material, which is available to authorized users.

## Background

Every year more than 230 million patients are scheduled for surgical procedures that require general anesthesia and mechanical ventilation [1], and these produce postoperative pulmonary complications (PPCs) that increase morbidity and mortality [2-7]. There are several reasons for the appearance of PPCs related to general anesthesia and mechanical ventilation: first, cyclic alveolar recruitment/derecruitment related to atelectasis, which appears in almost all patients during general anesthesia; second, alveolar overdistension related to the use of high tidal volume (VT) or inadequate positive end-expiratory pressure (PEEP) levels [8,9]. Even for short time periods, both these factors increase the alveolar and systemic inflammatory response, thus favoring lung injury [10,11] and multiple-organ system failure [12].

Protective ventilation with a low VT and adequate PEEP prevents lung injury and decreases morbi-mortality in critically ill patients with [13-15] or without [16] previous lung injury; this has also been shown for short-term intraoperative mechanical ventilation [17], as supported by evidence from two separate meta-analyses [18,19]. An intraoperative VT of 6–8 ml/kg, together with a PEEP of 6–8 cmH_2_O, decreases PPCs, readmissions, intensive care unit (ICU) length of stay (LOS), and even mortality, compared to ventilation with a VT of 10 ml/kg and a PEEP of 3 cmH_2_O [19]. Despite a significant decrease in the number of PPCs, they are still high in intermediate- and high-risk patients [18-20]. We hypothesize that this may be either because one standard protective strategy does not fit all patients or because a global approach, including a combined intraoperative and postoperative strategy, is required to minimize postoperative complications in these patients.

Lung injury has also been attributed to a high fraction of inspired oxygen (FiO_2_) by, among other phenomena, increasing the number of reactive oxygen species (ROS), which has raised concerns about the potential harm of perioperative hyperoxia [21]. In spite of this, we propose the use of 0.8 FiO_2_ intraoperatively for optimal care for several reasons. First, even though some studies have suggested that a high VT and hyperoxia have a synergistic effect that accentuates alveolar damage, lung-protective ventilation may substantially reduce additional risks from hyperoxia. The use of PEEP as well as protective mechanical ventilation (MV) has substantially reduced the risk related to hyperoxia for the vast majority of patients requiring MV [22]. Second, tissue hyperoxia decreases oxidative stress related to perioperative ischemia/reperfusion phenomena, an effect that has been demonstrated in colon, thoracic, and cardiac surgery [23]. Third, the use of a FiO_2_ of 0.8 during anesthesia has been shown to reduce surgical site infections (SSIs) in many surgical procedures [24], with an SSI risk reduction of 23% for all surgeries combined [25], and therefore the rationale for proposing hyperoxia as a factor in preventing SSIs is well established [26]. Finally, hyperoxia does not seem to increase atelectasis or other pulmonary complications after surgical procedures [24].

Complementary strategies for lowering VT and PEEP during the intra- and postoperative periods, such as alveolar recruitment maneuvers (ARMs), individualizing PEEP settings (through a decremental PEEP trial), FiO_2_ 0.8, and ventilatory support in the immediate postoperative period, have physiological benefits, such as improvements in oxygenation, ventilation and respiratory mechanics as well as and a reduction in PPCs [17,18,27-30]. However, these strategies are not commonly used together in clinical practice [30,31]. We hypothesized that, compared to a standard low-VT lung-protective ventilation strategy applied to all intermediate- to high-risk surgical patients, individualized ventilatory management consisting of a strategy for minimizing lung collapse and overdistension that combines the use of low VT, ARMs, an individualized PEEP trial, reevaluation of PEEP during the intraoperative period, and individualized ventilatory support in the postoperative period, will decrease postoperative pulmonary and systemic complications in patients with no previous lung injury.

Therefore, iPROVE aims to compare the efficacy of perioperative individualized ventilation and standard lung-protective ventilatory strategies to reduce the overall incidence of a composite of pulmonary and systemic complications.

## Methods

### Study design

**Figure 1:**
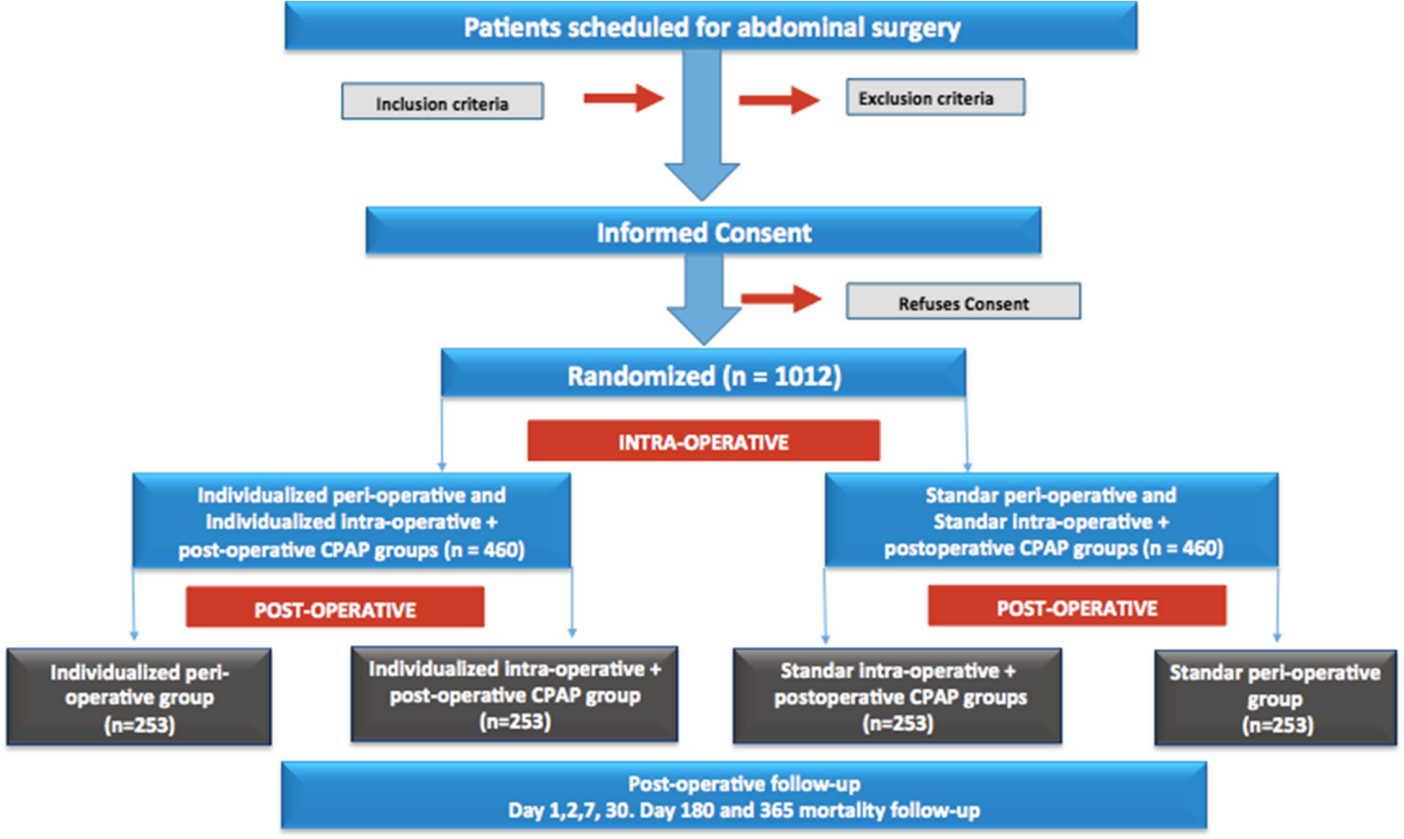
Flow diagram of iPROVE. *CPAP* continuous positive airway pressure.

The Individualized Perioperative Open-lung Ventilatory Strategy (iPROVE) trial is a comparative, prospective, multicenter, randomized, and controlled four-arm trial that will include 1012 patients (Figure 1).

The trial has been designed in accordance with the fundamental principles established in the Declaration of Helsinki, the Convention of the European Council relating to human rights and biomedicine, and the Universal Declaration of UNESCO on the human genome and human rights, and with the requirements established by Spanish legislation in the field of biomedical research, the protection of personal data, and bioethics, which was classified by the Spanish Agency of Drugs and Medical Devices as a clinical randomized study without drugs on 8 April 2014 and registered on 5 June 2014 at http://www.clinicaltrials.gov with identification no. NCT02158923. Approval of the final protocol by the local committee at each participation center has been obtained prior to recruitment initiation (see Additional file 1).

### Study population

The inclusion criteria of the study population consists of male and female patients ≥18 years old, with an intermediate or high risk of PPCs as defined by the ARISCAT score (based on the analysis of seven factors, in which a score between 26 and 44 points defines an intermediate risk and a score of more than 44 points defines a high risk [31]), with a body mass index (BMI) of <35 kg/m^2^, who are scheduled for major abdominal (laparotomy and laparoscopic) surgery with an expected operating time of more than 2 h (see Additional file 2). Exclusion criteria are age <18 years, pregnancy or breast-feeding, a body mass index of >35 kg/m^2^, moderate or severe acute respiratory distress syndrome (ARDS; PaO_2_/FiO_2_ < 200 mmHg), diagnosis of heart failure defined as a cardiac index <2.5 ml/min/m^2^ or >2.5 when ≥5 μg/kg/min dobutamine is required, or suspicion of heart failure according to clinical signs (hypotension, oliguria, pulmonary edema) together with NT-proBNP >13 pg/ml, diagnosis or suspicion of intracranial hypertension (>15 mmHg), mechanical ventilation in the last 15 days (including CPAP), presence of pneumothorax or giant bullae on a chest radiograph or computed tomography (CT), patients with chronic obstructive pulmonary disease (COPD) requiring oxygen or CPAP, and patients participating in another interventional study.

### Method of randomization and bias minimization

Informed consent will be obtained from each participant before enrollment in the study. Patients who meet all the inclusion criteria and none of the exclusion criteria will be consecutively included and randomized into one of the four study arms (Figure 1):

1. Individualized perioperative group;
2. Individualized intraoperative group + postoperative CPAP;
3. Standard intraoperative group + postoperative CPAP;
4. Standard perioperative group.

The patients will be randomized online via the website http://iprove.incliva.es using the Mersenne Twister algorithm with an allocation rate of 1:1:1:1.

Blinding: At least two investigators are required in each participating center, because the study characteristics do not allow the blinding of investigators in the operating and postoperative room, so data acquired in these sites will not be blinded. After 24 h, all data will be acquired by the second investigator who will be blinded to the randomization arm.

### General procedures

All participating patients, regardless of the study arm into which they are randomized, will be monitored and managed following general standard of care practices aimed at maintaining optimal conditions. Both intraoperative and immediate-postoperative (3 h) anesthetic management (unrelated to ventilatory management) will be decided by the attending physician as they see fit, following the established protocols at each center. However, in order to ensure a high standard of anesthetic management, a number of common strategies have been established: halogenated agents will be given to maintain anesthesia, intra- and postoperative pain will be controlled with neuraxial anesthetics, hemodynamic management will be based on advance cardiac output monitoring, and fluids will be administered following goal-direct therapy principles. Appropriate antibiotic prophylaxis will be administered, and pharmacological prevention of postoperative nausea and vomiting (PONV) will be adopted. Finally, when nasogastric tube insertion is required, it should be withdrawn prior to extubation when possible. All these data will be collected and analyzed.

### Monitoring

Intraoperative monitoring will include an electrocardiogram (ECG), pulse oximetry, capnography, bladder or esophageal temperature, anesthetic depth analysis (bispectral analysis, BIS) and a neuromuscular blockade (with train of four, TOF), invasive blood pressure measurements, and advanced hemodynamic monitoring with minimally invasive monitoring (optional depending on the standard clinical practice and availability of equipment at each hospital). Ventilatory parameters will be monitored by the anesthesia machine: VT, PEEP, FiO_2_, peak airway pressure (P_aw_), plateau pressure (P_plat_), and dynamic compliance of the respiratory system (C_rs_). Intra-abdominal pressure (IAP) will be monitored during laparoscopic surgery. Postoperative monitoring will include at least an ECG, pulse oximetry, and invasive arterial pressure measurements.

### General intraoperative ventilator management

Pre-oxygenation will be performed for 5 min at FiO_2_ 0.8 with a tightly sealed face mask before induction. Patients will be ventilated in volume control mode (VCV) with squared flow, a VT of 8 ml/kg of the predicted body weight (PBW), and a P_plat_ of ≤25 cmH_2_O. If the P_plat_ reaches or exceeds 25 cmH_2_O, VT will be decreased in 1 ml/kg steps until the P_plat_ drops to ≤25 cmH_2_O. The respiratory rate (RR) will be set to maintain an end-tidal carbon dioxide partial pressure (EtCO_2_) between 35–45 mmHg, with an inspiratory to expiratory ratio (I:E) of 1:2 and a inspiratory pause time of 20% of the inspiratory time. FiO_2_ will be set at 0.8 throughout the whole procedure. During the awakening period from general anesthesia (patients with spontaneous ventilation), a FiO_2_ of 0.8 will be applied at the same end-expiratory pressure used, using either PEEP or CPAP.

Extubation will not be allowed by applying a positive pressure above the previously set PEEP or CPAP or while suctioning through the tracheal device. If necessary, aspiration can be performed at least 10 min before extubation. After suction, the patient will be switched back to mechanical ventilation. If the patient is randomized into the individualized perioperative or individualized intraoperative + postoperative CPAP group, a new alveolar recruitment maneuver will be performed. Once extubation has been performed, the patient will be oxygenated with 0.5 FiO_2_ through a Venturi mask.

### Specific intraoperative ventilatory management

The intraoperative ventilatory management comprises a two-arm management strategy (Figure 1).

#### Standard group

The patients will be ventilated as previously described in the General intraoperative ventilator management section.

#### Individualized group

In this group, an ARM is performed after intubation followed by a PEEP titration trial. Before the recruitment is performed the anesthesiologist must ensure that there is hemodynamic stability [mean arterial pressure (MAP) of more than 70 mmHg and/or a cardiac index of more than 2.5 ml/min/m^2^] for at least 5 min, a stroke volume variation (SVV) of less than 10%, and an adequate neuromuscular blockade (0 of 4 by TOF). The ARM is performed as described in the following section.

### Alveolar recruitment maneuver (ARM)

**Figure 2:**
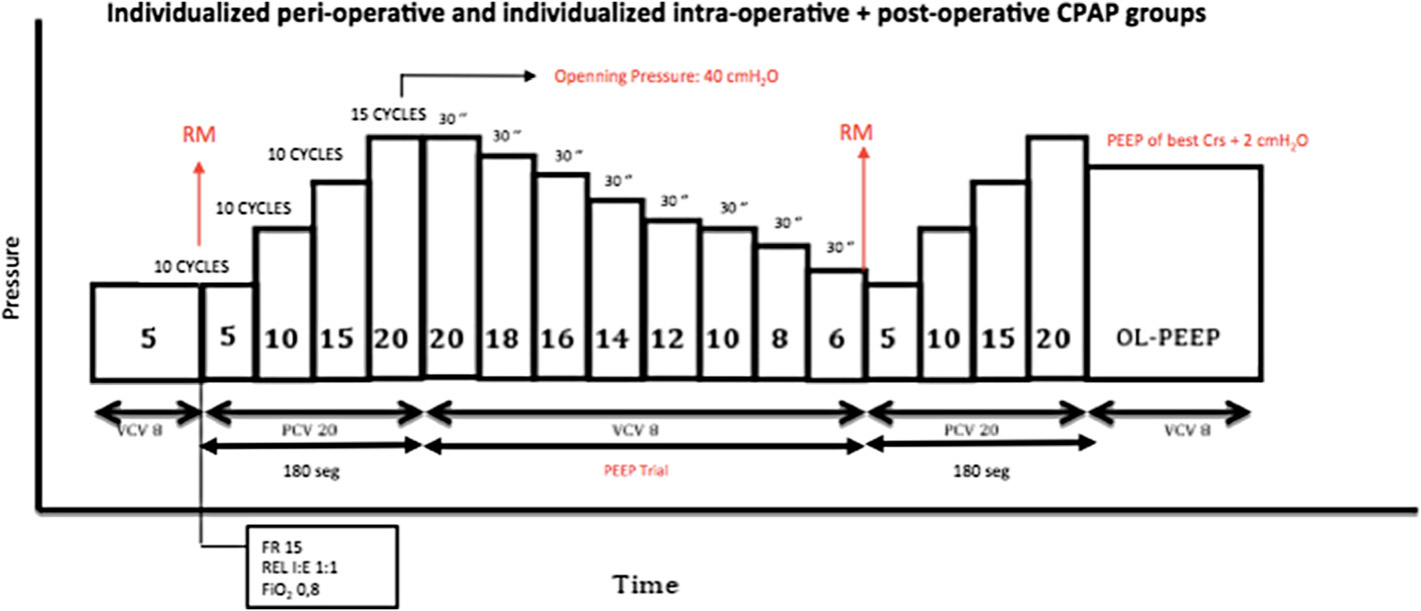
Alveolar recruitment maneuver and PEEP titration trial protocol. *RM* recruitment maneuver, *Crs* respiratory system compliance, *PEEP* positive end-expiratory pressure, *CPAP* continuous positive end-expiratory pressure, *VCV* volume-controlled ventilation, *PCV* pressure-controlled ventilation, *RR* respiratory rate, *I:E* inspiratory-to-expiratory ratio.

The ventilator will be changed from VCV to pressure-controlled ventilation (PCV) with a 20-cmH_2_O driving pressure and an RR of 15 breaths per minute (rpm), I:E of 1:1, 0.8 FiO_2_, and PEEP of 5 cmH_2_O. For the recruitment phase, the PEEP level will be increased in steps of 5 cmH_2_O every ten respiratory cycles, up to a PEEP of 20 cmH_2_O, to produce an airway opening pressure of 40 cmH_2_O and maintained for 15 respiratory cycles in the opening pressure [32] (total maneuver time: 180 s). If hemodynamic instability appears during the recruitment phase (a >50% decrease in the cardiac index or MAP), the maneuver will be interrupted and 5–15 mg ephedrine or 0.05-0.15 mg phenylephrine given; after hemodynamic stabilization, a new ARM will be performed. After lung recruitment is accomplished, the optimal PEEP is titrated through a decremental PEEP trial, as described in the following section [8] (Figure 2).

### Titration of the optimal individual positive end-expiratory pressure: Decremental PEEP trial

At the end of the last step of the PCV recruitment phase when the PEEP is 20 cmH_2_O, the mode will be switched to VCV with a VT of 8 ml/kg, RR of 15 rpm, and I:E of 1:2, 0.8 FiO_2_. After this, PEEP is decreased 2 cmH_2_O steps every 30 s until the highest C_rs_ observed on the ventilator’s monitor (until C_rs_ starts decreasing or does not increase). Once the best C_rs_ is known, a new recruitment maneuver is performed and the PEEP for the best C_rs_ + 2 cmH_2_O is adjusted. In the case of accidental airway depressurization, a new ARM is performed while an identical PEEP is set (Figure 2).

The need for new recruitment maneuvers and a PEEP trial is evaluated every 40 min by measuring the C_RS_ and peripheral capillary oxygen saturation (SpO_2_). If there is a drop of more than 10% of the C_rs_, FiO_2_ will be transitorily decreased to 0.21-0.25 for at least 4 min, and if SpO_2_ drops to ≤96% at this FiO_2_, a new recruitment and PEEP trial are performed.

### Intraoperative rescue maneuvers

In the case of arterial hypoxemia (SpO_2_ of ≤95% with FiO_2_ 0.8), after excluding endobronchial tube displacement, bronchospasm, pneumothorax, or a hemodynamic cause, a protocol for rescue therapy has been devised for each specific group.

#### Individualized group

A new recruitment maneuver and PEEP trial are performed. If SpO_2_ is less than 95%, FiO_2_ is increased in 0.1 steps.

#### Standard group

The 0.1 FiO_2_ is increased until SpO_2_ is more than 95%. If arterial hypoxemia persists with 1.0 FiO_2_, the PEEP is increased in steps of 2 cmH_2_O (until a maximum of 12 cmH_2_O).

### General postoperative management in the postoperative care unit

General postoperative management in the postoperative care unit (PACU) not related to ventilator management will be decided by the attending physician following the established protocols at each center. Patients will be oxygenated with FiO_2_ 0.5 through a Venturi mask for the first 15 min. The arterial oxygenation will be evaluated 15 to 30 min later when patients are awake and collaborative [Glasgow coma score (GCS) higher than 13] without any residual anesthetic effect (Richmond scale −1 to +1) and under pain control [verbal analog pain scale (*echelle verbal analogique*; EVA) score <4] by decreasing the FiO_2_ to 0.21 for at least 5 min (air test). The air test is intended to identify possible decreases in SpO_2_ related to postoperative atelectasis that may have been masked by the use of 0.5 FiO_2_. The air test is not performed if the patient already has an SpO_2_ below 96% with FiO_2_ 0.5. Air tests are repeated at 60, 120, and 180 min after PACU admission in all study patients. When the patient arrives in the PACU or ICU with invasive mechanical ventilation, the above-mentioned management will be applied after extubation.

### Specific postoperative ventilatory management

Postoperative ventilatory management consists of two types of management for each of the two intraoperative arms (Figure 1).

#### Individualized perioperative group

Supplemental oxygen at FiO_2_ 0.5 will be delivered through a Venturi mask. If SpO_2_ falls below 96% during the room-air test (FiO_2_ 0.21 for 5 min), a CPAP of 5 cmH_2_O (or 10 cmH_2_O if the BMI exceeds 30) with FiO_2_ 0.5 will be initiated. Reevaluation of arterial oxygenation will be performed every hour for the first 3 postoperative hours. If patients require CPAP at any time during their PACU stay, it will be maintained for 3 h, independently of their oxygenation levels.

#### Individualized intraoperative + postoperative CPAP group

A CPAP of 5 cmH_2_O (or 10 cmH_2_O if the BMI exceeds 30) with a FiO_2_ 0.5 will be applied to all patients in this group.

#### Standard + postoperative CPAP group

A CPAP of 5 cmH_2_O (or 10 cmH_2_O if the BMI exceeds 30) with a FiO_2_ 0.5 will be applied to all patients in this group.

#### Standard group

Supplemental oxygen at 0.5 FiO_2_ will be delivered through a Venturi mask.

If any patient in any group experiences PONV or does not tolerate the CPAP device, they will be changed to standard therapy.

### Postoperative rescue maneuver

Rescue therapies are initiated if the SpO_2_ decreases to 92% or less during the air test or if SpO_2_ is 95% or less while on 0.5 FiO_2_, with or without pressurization according to the patient’s randomized group. Evaluation of a positive or negative response to the rescue maneuver is performed in a maximum period of 30 min.

Individualized perioperative group:

1. For patients with a 0.5 FiO_2_ Venturi mask, the rescue maneuver is started with 5 cmH_2_O CPAP (or 10 cmH_2_O if the BMI exceeds 30). If the patient is already on 5 cmH_2_O CPAP, it will be increased to 10 cmH_2_O.
2. In patients with a BMI exceeding 30 or with persistent hypoxemia and/or hypercapnia [blood partial pressure of carbon dioxide (PaCO_2_) >50 mmHg with a pH <7.30], tachypnea (RR >25 rpm), or increased activity of accessory respiratory muscles are present, inspiratory support with noninvasive ventilation (NIV) will be started.

Individualized intraoperative + postoperative CPAP and Standard + postoperative CPAP group:

1. CPAP will be increased to 10 cmH_2_O in all cases.
2. In patients with a BMI of more than 30 or persistent hypoxemia and/or hypercapnia (PaCO_2_ > 50 mmHg with a pH <7.30), tachypnea (RR >5 rpm), or increased activity of accessory respiratory muscles are present, inspiratory support with NIV will be started.

Standard group:

1. FiO_2_ will be increased to 0.8 through a controlled FiO_2_ mask with a reservoir bag.
2. If there is persistent hypoxemia, CPAP of 5 cmH_2_O (or 10 cmH_2_O if the BMI exceeds 30) will be used.
3. If hypoxemia persists, CPAP will be increased to 10 cmH_2_O.
4. If persistent hypoxemia and/or hypercapnia (PaCO_2_ > 50 mmHg with a pH <7.30), tachypnea (RR exceeding 25 rpm), or increased activity of accessory respiratory muscles are present, inspiratory support with NIV will be started.

### Noninvasive ventilation (NIV)

The ventilator (specific for NIV or with software for NIV) and interface for NIV will be chosen by the attending physician and based on hospital availability. CPAP, i.e., expiratory positive airway pressure (EPAP), and FiO_2_ will be set according to the patient’s randomized group. Positive pressure will start with an inspiratory positive airway pressure (IPAP) of 5 cmH_2_O higher than the EPAP and will be increased in steps of 5 cmH_2_O up to 15 cmH_2_O. The EPAP will be increased to a maximum of 10 cmH_2_O (15 cmH_2_O if the BMI exceeds 30).

### Invasive ventilation

Direct tracheal intubation (without NIV trial) will be indicated if the patients also meet at least one of the following criteria:

1. Hemodynamic instability [a systolic blood pressure (SBP) <80 mmHg or <40% of the basal or vasoactive drug requirements for more than 2 h is required to maintain the SBP above 80 mmHg].
2. Ventricular arrhythmias with hemodynamic instability or ECG signs of myocardial ischemia.
3. GCS of less than 9.
4. Sedation requirement due to agitation.

Tracheal intubation after 1 h of NIV will be indicated in patients meeting at least one of the following criteria:

1. Severe hypoxemia (SpO_2_ < 92% with a FiO_2_ as prescribed according to the randomized group).
2. Respiratory acidosis (pH <7.30 with a PaCO_2_ > 50 mmHg).
3. Signs of distress with increased use of accessory respiratory muscles or paradoxical thoracic-abdominal respiratory movements.

### Study variables

The primary outcome of the iPROVE trial is a composite of pulmonary and systemic complications experienced by the study population in the first 7 days after surgery and can be divided into respiratory complications and systemic complications as discussed below.

Respiratory complications:

1. Atelectasis: defined by a combination of SpO_2_ ≤ 96% during the air test and chest X-ray images suggesting lung opacities with a shift in the mediastinum, hilum, or hemidiaphragm toward the affected area and compensatory overinflation in the adjacent non-atelectic lung.
2. Hypoxemia: defined as SpO_2_ of 92% or less with 0.21 FiO_2_ or SpO_2_ of 95% or less with 0.5 FiO_2_.
3. ARDS: according to the Berlin definition [33].
4. Pneumonia: the presence of a new pulmonary infiltrate and/or progression of previous pulmonary infiltrates on a chest radiograph plus at least two of the following criteria: (a) leukocytosis with >12,000 WBC/mm^3^ or leukopenia with <4000 WBC/mm^3^, (b) fever >38.5°C or hypothermia <36°C, and (c) increased secretions with purulent sputum and a positive bronchial aspirate.
5. Pleural effusion: chest x-ray with the presence of costophrenic angle blunting, displacement of adjacent anatomical structures, and blunting of the hemidiaphragmatic silhouette in the supine position.
6. Bronchospasm: presence of expiratory wheezing treated with bronchodilators.
7. Pneumothorax: air in the pleural space and the mediastinum is shifted to the opposite side (a thorax radiography will be performed in suspected cases of auscultation hoarseness).
8. Aspiration pneumonitis: respiratory failure after the inhalation of regurgitated contents.
9. Requirements for rescue maneuvers: increased FiO_2_, increased requirement for CPAP, or the need for noninvasive or invasive ventilation.
10. Early extubation failure or requirement of reintubation.

Systemic complications:

1. Heart failure: cardiac index <2.5 ml/min/m^2^ or >2.5 when ≥5 μg/kg/min dobutamine is required. Clinical signs (hypotension, oliguria, pulmonary edema) together with NT-proBNP >13 pg/ml or echocardiographic diagnosis [34].
2. Systemic inflammatory response syndrome (SIRS): axillary temperature >38.5°C or <35.5°C, or central temperature of 38°C and 36°C, respectively. Heart rate (HR) >90 bpm (in the presence of atrial arrhythmia, the ventricular rate measurement will be used). If medication that could affect the HR is administered, the patient must meet the following three criteria: (a) RR >20 rpm, (b) a PaCO_2_ of <32 mmHg or use of mechanical ventilation, (c) leukocytosis of ≥12 × 10^9^/l, or leukopenia of <4 × 10^9^/l.
3. Sepsis: infectious focus identified plus SIRS criteria.
4. Severe sepsis: sepsis plus at least one organ dysfunction, hypoperfusion, or hypotension.
5. Septic shock: Severe sepsis with hypotension and hypoperfusion that is unresponsive to fluids.
6. Renal failure, following the acute kidney injury scale [35].
7. Anastomosis dehiscence: suture line failure with leakage of the intraluminal contents that may cause peritonitis, fistula from the wound or drain, or appearing as an abdominal fluid collection (diagnosed with imaging) that causes fever, septicemia, and shock.
8. Abdominal abscess diagnosed with imaging techniques (CT).
9. Surgical wound infection: following the CDC criteria [36].
10. Surgical reintervention required.

The secondary outcomes are the composite of postoperative pulmonary complications at 7 days and over the first 30 post-surgical days. Other secondary outcomes are:

1. Increased ICU length of stay (LOS).
2. Increased hospital LOS.
3. ICU readmission in the first 30 days after surgery.
4. Hospital readmission in the first 30 days after surgery.
5. Mortality within 30, 180, or 365 days.
6. The presence of plasma inflammatory markers: increased expression of interleukin 8 (IL-8), tumor necrosis factor alpha (TNF-α), and monocyte chemo-attractant protein (MCP-1). Plasma samples will be taken preoperatively and 48 h post-surgery.

The primary and secondary data outcomes will be taken 15 min and 3 h after PACU/ICU admission and at 1, 2, 7, and 30 days after surgery, with a 180- and 365-day follow-up for mortality. Plasma samples will be taken preoperatively and at 2 days after surgery. If the patient is not extubated in the operating room, the first four data time points will be taken from the time of extubation.

### Other follow-up variables

Baseline variables will be recorded preoperatively and are age, sex, height, weight, body mass index, American Society of Anesthesiologists (ASA) physical status, sequential organ failure assessment (SOFA) score, ARISCAT [26] risk score, type of intervention, and medical history.

Intraoperative parameters recorded at three different time points (post-induction, 60 min after induction, and pre-extubation) will be: arterial blood gases, SpO_2_, FiO_2_, respiratory variables [VT, PEEP, P_aw_, P_plat_, C_rs_, respiratory system resistance (R_aw_), hemodynamics (cardiac index, PAM, and stroke volume variation (SVV) and/or pulse pressure variation (PPV)], diuresis, and temperature. Other relevant data that include the types of anesthetic drugs used, type and volume of fluids, blood loss and transfusion requirements, need of vasoactive drugs, diuresis, nasogastric tube insertion, duration of surgery, mechanical ventilation time, number of recruitment maneuvers performed, and the need for rescue therapy will also be recorded.

### Statistical analysis

The sample size was estimated from the literature, assuming a risk of 25% for developing postoperative pulmonary or non-pulmonary complications [17], a relative reduction of 50% in the incidence of these complications with individualized alternative treatment in the groups, and taking into account the statistical power for making matched comparisons between the four groups in the study [37]; using a significance level of 5% and a power of 80% results in a total requirement of 920 patients (230 in each ventilatory management group). This figure was enlarged to 1012 (10%) to compensate for possible dropouts. The recruitment among centers will be competitive.

First, the patient baseline variables will be described and the homogeneity of these groups evaluated using appropriate statistical tests for the type of variable being analyzed (mean difference of proportions, chi-square, ANOVA, with a corresponding confidence interval of 95%). Then bivariate associations between patient characteristics, the primary endpoint, and the secondary outcomes will be performed by calculating their respective odds ratios or, in the case of a quantitative outcome, by an ANOVA test.

Following this, the association between the intervention groups and the main and secondary outcomes will be analyzed, calculating the corresponding odds ratio, or ANOVA in the case of quantitative outcomes. In all cases, respective means or proportions are estimated with their respective 95% confidence intervals. The primary outcome, pulmonary complications outcome, non-pulmonary complications, and mortality measurement analyses will be repeated using multivariate logistic regression models and adjusted to any patient characteristics that are shown to be clinically relevant by the previous bivariate analyses. Similarly, a multilevel analysis will be performed, incorporating different hospitals as random effects in order to assess whether the recruitment center influenced the results.

The monitoring plan is based on the modified Haybittle-Peto boundaries for stopping trials after interim analyses in the second half of the inclusion period [38,39]. Analysis of the main endpoint will be presented to the Data and Safety management board under a blinded code for allocation group. The first interim analysis will be conducted when outcome data of 460 trial participants have been obtained and/or the main endpoints for 150 participants have been documented. If this first interim analysis is significant (*P* <0.001) for benefit or harm from the intervention, a second interim analysis will be carried out when outcome data for 600 trial participants have been obtained. If this second interim analysis is also statistically significant (*P* <0.001) for benefit or harm, the Data and Safety management board will advise the Steering Committee to stop the trial.

### Trial organization

The steering committee is constituted by the study principal investigators who contributed to its design and approved the final protocol. The executive committee comprises the main investigators of each participating center and is responsible for administrative, trial, and data management. The Data and Safety management board is composed of independent experts in mechanical ventilation and multicenter trials, and it recommends the continuance or discontinuation of the study based on the evidence collected at interim analysis intervals. The trial management team comprises a chief investigator, a project manager, a statistician, and an investigator expert in informatics. The responsibilities of this team are:

1. Planning and conducting the study: designing the protocol, case report, and electronic case report (e-CRF) forms, designing the investigator manual, and managing and controlling the data quality.
2. Research center support: assisting the centers with the administrative submission, monitoring recruitment rates and taking action to increase recruitment if necessary, monitoring follow-ups, auditing, and sending study materials to the research centers.
3. Producing a monthly study newsletter and developing supporting material for the study.
4. Statistical analysis and research reporting: complete statistical analysis and helping in writing the final manuscript.

## Discussion

Postoperative pulmonary and systemic complications are a common problem in patients with an intermediate or high risk of PPCs, and there is clear evidence showing that protective mechanical ventilation can attenuate these complications by limiting lung injury and the systemic inflammatory response [17,20].

Atelectasis appears in almost all patients during general anesthesia, favoring the two mechanisms of ventilator-induced lung injury (VILI) [40-42]: alveolar cyclic recruitment/derecruitment and overdistension because the aerated part of the lung receives most of the VT. These mechanisms produce alveolar damage and trigger the local and systemic inflammatory responses, favoring pulmonary and systemic lung complications, even in short-term mechanical ventilation in previously healthy lungs [12,43].

Lung-protective mechanical ventilation with a low VT minimizes overdistension and reduces lung injury not only in ARDS patients or patients without lung injury receiving long-term mechanical ventilation, but also during intraoperative short-term mechanical ventilation. This has been recently confirmed in a randomized controlled trial [17] and in previous meta-analyses [18,19]. The use of a low VT favors atelectasis and therefore makes PEEP the other key point in the lung-protective ventilatory strategy, which aims to avoid cyclic recruitment/derecruitment. However, the ideal PEEP level is not yet known because of the high heterogeneity in the methods for setting the PEEP settings and the different PEEP levels used in previous trials.

Recently, two large randomized controlled trials tried to show the benefits of the open lung concept, including recruitment maneuvers and higher PEEP levels than those normally used in clinical practice. The PROtective Ventilation using High versus Low positive end-expiratory pressure (PROVHILO) study showed no differences between *high* PEEP (12 cmH_2_O) levels combined with recruitment maneuvers compared to a *low* PEEP (less than 2 cmH_2_O) levels [20]. These results may be explained by the use of an inadequate opening pressure during the recruitment maneuver (30–35 cmH_2_O) during only three respiratory cycles or may be related to the use of inadequately high PEEP levels that may well be as harmful in terms of lung injury as low PEEP levels. The Intraoperative Protective Ventilation in Abdominal Surgery (IMPROVE) study showed that the strategy of a VT of 6–8 ml/kg IBW with repetitive recruitment maneuvers combined with a standardized PEEP level (6–8 cmH_2_O) was beneficial, but only compared to the nonprotective ventilation strategy using high VT (10–12 ml/kg IBW) with no PEEP [17].

Although the IMPROVE strategy resulted in a positive effect of the open lung concept, we believe that it could be improved by limiting the unnecessary number of recruitment maneuvers during the intraoperative period because it is known that ARMs themselves can trigger an inflammatory response. Thus, this strategy can be further improved by individualizing the PEEP level titration; this approach maintains the benefits of the maneuver and makes it more time-effective, therefore decreasing the need for repeated recruitment maneuvers [8]. Another important intraoperative strategy for optimal care proposed in this study is the use of 80% supplemental oxygen, which has been demonstrated to reduce the incidence of SSIs without increasing postoperative pulmonary complications [24]. Finally, atelectasis formation is favored in the immediate postoperative period by many factors. Several clinical studies show that prophylactic pressurization, or pressurization at the first sign of hypoxemia, reduces postoperative pulmonary complications [30].

Despite these interesting results, there are no clinical trials that apply these strategies with an individualized approach in both the intraoperative and postoperative period settings, i.e., by integrating intraoperative and immediate postoperative strategies, but aiming all of them toward protecting the lung from injury and thus reducing pulmonary and non-pulmonary postoperative complications.

In this trial, the effectiveness of individualized implementation and global approaches to lung-protective ventilation, to keep lung collapse and overdistension to a minimum, will be evaluated. The strategy includes all the maneuvers that have previously been shown to have a beneficial effect:

1. Low VT
2. ARMs
3. Individualized PEEP setting with a decremental PEEP trial
4. Reevaluation of PEEP during the intraoperative period
5. High intraoperative FiO_2_
6. Individualized ventilatory support in the postoperative period.

If the trial demonstrates that the individualized perioperative global approach decreases postoperative complications, these findings will represent a big improvement in the management of moderate- and high-risk surgical patients.

## Trial status

The iPROVE screening for patients begins in January 2015. Local ethics approval at each participation center is required.

## Appendix 1

The iPROVE Investigators consist of:

Steering committee: Carlos Ferrando, Javier Belda, Marina Soro, Jaume Canet, Carmen Unzueta, Fernando Suárez, Julián Librero, Alicia Llombart.

Executive committee: Carlos Ferrando, Jaume Canet, Mª Carmen Unzueta, Lucas Rovira, Fernando Ramasco, Manuel Granell, César Aldecoa, Oscar Diaz, Jaume Balust, Ignacio Garutti, Roque Company, Teresa Alonso, Rafael Gonzalez, Mª Eugenia Durán, Lucia Gallego, Santiago García del Valle, Javier Redondo, Pedro Diaz, David Pestaña, Aurelio Rodríguez, Marisol Hernandez, Javier García, Elena Espinosa, Pedro Charco, Manuel de la Matta, Maite Ibáñez, Francisco Barrios.

Data and Safety management board: Jesus Villar, Joao Borges, Samir Jaber.

Trial management committee: Carlos Ferrando, Alicia Llombart, Marina Soro, Carlos Delgado, Salvador Peiró.

## Appendix 2

iPROVE investigators:

Esther Romero, Carolina Romero, Amanda Miñana, Tania Moreno, Antonio Katime, Estefanía Gracia, Ana Izquierdo, Tania Socorro, Concepción Rubio, Paola Valls, Angels Lozano, Alejandro Duca, Raul Incertis, Isabel Fuentes, Ana Jurado, Juan Carrizo, Jose Navarro, Abigail Villena, Ferran Serralta, Jose A Carbonell, Jaume Puig, Ernesto Pastor, Blanca Arocas, Mª Luisa García, Andrea Gutierrez, Gerardo Aguilar, Ana Mugarra, Jose M. Alonso, Maria J. Parra, Mario de Fez, Esperanza Mata, Jesus Nieves, Carlos Alvarez, Raquel Tolos, Mar Sendra, Andrea Brunelli, Virginia Cegarra, Mercedes García, Gonzalo Azparren, Patricia Piñeiro, AnaM Lajara, Jose M Pérez, Jose A de Andrés, Maria J Hernández, Lorena Gómez, Teresa Alonso, Sara Rodiño, Marta López, AnaM Pérez, Jose M Marcos, Fernando Díez, Mª Piedad Martínez, Mª del Mar Hernández, José Fernandez-Pacheco, Maria J Rivera, M Galiana, Roque Company, Ana Colás, Irene Molinós, Ana Asensio, Margarita Vergas, Clara García, Jesus Rico, Pablo García, Jose I. García, Viviana Varón, Eva Romero, Guido Mazzinari, Esperanza Herrera, Eva Rosado, Patricia Ramos, Nazario Ojeda, Oto Padrón, Lucia Valencia, Sergio Cabrera, Rayco Rodríguez, Antonio Romero, Ana González, Jessica García, Nilda Martinez de Castro, Cristina Medrano, Nuria Mané, Graciela Martínez, Roger Pujol, Amalia Alcon, Vicente Torres, Javier Román Marta García, Alejandro Dominguez, Inmaculada Benítez, Domingo González, Daniel López-Herrera, M. Sol Hernández, Clara Morales, David Soriano, David Domínguez, Samuel Hernández, Maria Vila, Maria J Alberola, Sandra Verdeguer, Maite Ibáñez, Elena Lozano, Jose Valdivia, Vicente Gilabert, Francisco Barrios, Mercedes Ayuso, Ricardo Moreno.

## Additional files

Additional file 1:
**Ethics committees that approved the final protocol.**
Additional file 2:
**Surgical procedures included in the study protocol.**

## Abbreviations

ARDS: Acute respiratory distress syndrome
ARM: Alveolar recruitment maneuvers
ASA: American Society of Anesthesiologists
BIS: Bispectral analysis
BMI: Body mass index
Bpm: Breaths per minute
C: Confidence interval
COPD: Chronic obstructive pulmonary disease
CPAP: Continuous positive airway pressure
C_rs_: Dynamic compliance of the respiratory system
CT: Computed tomography
ECG: Electrocardiogram
EPAP: Expiratory positive airway pressure
EtCO_2_: End-tidal carbon dioxide partial pressure
EVA: Visual analog pain scale (*echelle visuelle analogique* EVA)
FiO_2_: Inspiratory oxygen fraction
GCS: Glasgow Coma Score
HR: Heart rate
ICU: Intensive care unit
I:E: Inspiratory-to-expiratory ratio
IL-8: Interleukin 8
IPAP: Inspiratory positive airway pressure
IPROVE: Individualized perioperative open lung ventilatory strategy
LOS: Length of stay
MAP: Mean arterial pressure
MCP-1: Monocyte chemo-attractant protein
MV: Mechanical ventilation
NIV: Noninvasive ventilation
PaCO_2_: Partial pressure of carbon dioxide
PACU: Postoperative care unit
P_aw_: Peak airway pressure
PBW: Predicted body weight
PCV: Pressure control ventilation
PEEP: Positive end-expiratory pressure
PPCs: Postoperative pulmonary complications
P_plat_: Plateau pressure
PONV: Postoperative nausea and vomiting
PPV: Pulse pressure variation
R_aw_: Respiratory system resistance
ROS: Reactive oxygen species
RR: Respiratory rate
SBP: Systolic blood pressure
SIRS: Systemic inflammatory response syndrome
SOFA: Sequential Organ Failure Assessment
SpO_2_: Peripheral capillary oxygen saturation
SSI: Surgical site infection
SVV: Stroke volume variation
TNF-α: Tumor necrosis factor alpha
TOF: Train of four
VCV: Volume control ventilation
VILI: Ventilator-induced lung injury
VT: Tidal volume
