# Supplementary material for: Rationale and study design for an individualized perioperative open lung ventilatory strategy (iPROVE): study protocol for a randomized controlled trial
Source: Trials. 2015 Apr 27;16:193. doi: 10.1186/s13063-015-0694-1 (PMC4425893; doi:10.1186/s13063-015-0694-1)
Supplement: Additional file 1: — Ethics committees that approved the final protocol. [file 13063_2015_694_MOESM1_ESM.docx]

**Ethical Committees:**

Comité Ético de Investigación Clínica (CEIC) of the Hospital Clínico Universitario of Valencia, Comité Ético de Investigación Clínica of the Hospital Germans Trias i Pujol (CEIC HUGTiP), Comité Ético de Investigación Clínica (CEIC) of the Hospital de la Santa Creu i Sant Pau, Comité Ético de Investigación Clínica (CEIC) of the Hospital U y P La Fe. This Committee also aproved the ethics documentation for the Hospital of Manises, Comité Ético de Investigación Clínica (CEIC) of the Hosptial Universitario La Princesa, Comité Ético de Investigación Clínica (CEIC) of the Consorcio Hospital General of Valencia, Comité Ético de Investigación Clínica (CEIC) of the Hospital Universitario Río Hortega, Comité Ético de Investigación Clínica (CEIC) of the Hospital Clinic de Barcelona, Comité Ético de Investigación Clínica (CEIC) of the Hospital Gregorio Marañon, Comité Ético de Investigación Clínica (CEIC) of the Hospital Universitario Virgen del Rocio, Comité de Ético de Investigación Clínica (CEIC) of the Complejo Universitario Juan Canalejo, Comité de Ético de Investigación Clínica (CEIC) of the Hospital de León, Comité Ético de Investigación de Investigación Clínica (CEIC) of the Hospital Universitario Virgen de la Arraixaca, Comité Ético de Investigación Clínica (CEIC) of the Hospital Miguel Servet, Comité Ético de Investigación Clínica (CEIC) of the Hospital Universitario Fundación of Alcorcón, Comité Ético de Investigación Clínica (CEIC) of the Hospital General of Ciudad Real, Comité Ético de Investigación Clínica (CEIC) of the Hospital Universitario Nuestra Señora de Valme, Comité Ético de Investigación Clínica (CEIC) of the Hospital Ramón y Cajal, Comité Ético de Investigación Clínica (CEIC) of the Hospital of Gran Canaria Dr. Negrín, Comité Ético de Investigación Clínica (CEIC) of the Hospital of Galdakano-Usansolo, Comité Ético de Investigación Clínica (CEIC) of the Complejo Hospitalario Juan Ramón Jimenez, Comité Ético de Investigación Clínica (CEIC) of the Hospital Puerta de Hierro, Comité Ético de Investigación Clínica (CEIC) of the Hospital Nuestra Señora de la Candelaria, Comité Ético de Investigación Clínica (CEIC) of the Hospital Son Espases, Comité Ético de Investigación Clínica (CEIC) of the Hospital General of Alicante. Comité Ético de Investigación Clínica (CEIC) of the Hospital Marina Baixa de Villajoyosa, Comité Ético de Investigación Clínica (CEIC) of the Hospital Universitario Principe de Asturias.
