# Supplementary material for: Rationale and study design for an individualized perioperative open lung ventilatory strategy (iPROVE): study protocol for a randomized controlled trial
Source: Trials. 2015 Apr 27;16:193. doi: 10.1186/s13063-015-0694-1 (PMC4425893; doi:10.1186/s13063-015-0694-1)
Supplement: Additional file 2: — Surgical procedures included in the study protocol. [file 13063_2015_694_MOESM2_ESM.docx]

**Surgical procedures included in the iPROVE trial:**

Laparoscopic or laparotomy, oncologic or non-oncologic surgery.

Duodenopancreatectomy, liver resection, colorectal surgery, gastrectomy, esophageal surgical procedures, peritoneal carcinomatosis surgery, radical prostatectomy, radical cystectomy, biliary surgery, abdominal hysterectomy, vascular, others.
